# Sustainability of evidence based interventions implemented in CDC’s colorectal cancer control program

**DOI:** 10.1186/s43058-025-00734-9

**Published:** 2025-07-09

**Authors:** Krishna P. Sharma, Amy DeGroff, Michele Beckman, Sun Juzhong, Coleman King Sallyann, Joseph Djenaba

**Affiliations:** Division of Cancer Prevention and Control, National Center for Chronic Disease Prevention and Health Promotion, Centers for Disease Control and Prevention (CDC), Atlanta, GA USA

**Keywords:** Evidence based interventions, Colorectal cancer screening, Screening champion, Sustainability

## Abstract

**Background:**

The CDC’s Colorectal Cancer Control Program (CRCCP) partners with health system clinics to implement evidence-based interventions (EBIs) to increase colorectal cancer (CRC) screening prevalence. The sustainability of those EBIs is critical for the long-term success and impact of the CRCCP. This paper examines various aspects of the sustainability of these EBIs, including the factors associated with sustainability.

**Method:**

We used Clinic Data collected by CDC for program evaluation. The study employed two definitions of sustainability and conducted a comprehensive analysis including all available information on sustainability in the Clinic Data. Our descriptive analysis included comparing frequencies and means of the outcome variable as defined in the study. Logistic regression methods were used to explore the association of multiple explanatory factors with EBI sustainability.

**Results:**

The results highlighted significant variations in the sustainability of different EBIs. Provider reminders were reported as sustainable by 82.0% of the clinics, while reducing structural barriers were reported as sustainable by 55.6% of the clinics. The percentage of clinics able to sustain each of the four EBIs trended upwards over time, ranging from 13 to 34 percentage points increase. Clinics that had implemented EBIs before CRCCP involvement, those that integrated multiple interventions, and those with dedicated screening champions were more likely to sustain EBIs in the long term.

**Conclusions:**

We found substantial improvement in the sustainability of EBIs over the 5-year program period, although results varied by EBIs and room for improvement remains. The findings offer valuable insights for future implementation and sustainability of EBIs.

Contributions to the literature

- Existing research largely addresses the theoretical and methodological aspects of sustainability of public health programs, leaving a significant gap in our understanding of sustainability based on the evidence from actual program implementation.
- The CDC's Colorectal Cancer Control Program effectively increases CRC screening, but the sustainability of various program components and the contributing factors remain underexplored.
- This study addresses these gaps by providing empirical data on the sustainability of evidence-based interventions (EBIs), offering valuable insights that can be contextualized to enhance program implementation and improve long-term sustainability.

## Background

Colorectal cancer (CRC) is the second leading cause of death from cancers affecting both men and women [1]. In 2020, there were 126,240 cases and 51,869 deaths from colorectal cancer [1, 2]. Screening can reduce the incidence and mortality from CRC by detecting and removing precancerous polyps and identifying CRC in its early stages when treatments are more effective [3]. The United States Preventive Services Task Force (USPSTF) recommends CRC screening for average risk adults age 45 to 75 years [4]. Yet, the most recent data available suggests only 7 in 10 (71.6%) of adults ages 50–75 are up to date with CRC screening. [5] Including adults aged 45–49, who were previously not recommended for screening, would likely result in a lower overall screening rate [6].

The Centers for Disease Control and Prevention’s (CDC) Colorectal Cancer Control Program (CRCCP) aims to reduce the burden of CRC by increasing screening prevalence in partnering clinics, particularly among populations experiencing health disparities [7]. Through a 5-year agreement, CDC funds 35 award recipients–including state health departments, tribes, universities, and other organizational types (e.g., health systems, healthcare organizations). To achieve the program goals, CRCCP recipients partner with clinics, such as federally qualified health centers (FQHCs), to newly implement or enhance evidence-based interventions (EBIs) and other strategies. These EBIs are also recommended by the Community Preventive Services Task Force in *The Community Guide* [8] and include client reminders, provider reminders, provider assessment and feedback, and reducing structural barriers. The CRCCP also supports patient navigation, a strategy more recently recommended in 2022.^7^ These EBIs and other strategies align with what is broadly referred to in the implementation science literature as *implementation strategies–*approaches that support effective service delivery, adoption, integration, and long-term sustainability of public health interventions within specific organizational contexts [9, 10] These strategies are referred to as EBIs in both the Community Guide and CRCCP, are based on their effectiveness as determined by a systematic review process, and work as implementation strategies to increase community demand, community access and provider delivery/referral [11]. We will also refer to them as EBIs hereafter in this article. While the CDC provides overall program guidance and recommends EBIs and other implementation strategies, health system have the flexibility to select and tailor interventions based on their specific needs and readiness. CRCCP recipients work closely with partner clinics, providing resources and technical assistance either directly or through affiliated organizations [12]. The first 5-year CRCCP project period using this intervention approach was funded from 2015–2020^6^; the current project period is 2020–2025. Given the competitive award process, the composition of recipients changed to some extent between the two project periods.

CDC conducts a robust evaluation of CRCCP concurrently with its implementation [7]. Studies evaluating the program cycle 2015–2020 reported substantial increases in clinic-level CRC screening rates, the primary outcome of interest [7, 13]. During the first three years of the program (2015 to 2018), clinics reported an average annual increase of 4.6 percentage points in screening [14]. In contrast, CRC screening rate in the general U.S. population increased by only 1.5 percentage points, from 67.3% to 68.8%, over a two-year period (2016 to 2018). [15, 16]Additionally, past studies have examined which EBIs, or combination of EBIs, as well as other implementation strategies (e.g., program champions), are effective in increasing CRC screening prevalence in clinics. Study results have consistently shown that these EBIs are associated with significant increases in clinic-level screening prevalence [13, 14]. These findings underscore the crucial role of EBIs, which form the foundation of the CRCCP, in driving clinic level improvements. Sustained implementation of these interventions is essential for maintaining the screening at higher level in the future.

Sustainability is a tenet of the CRCCP [7]. Our underlying assumption is that if EBI implementation is sustained, the increased screening prevalence is also sustained, at least to some extent, even after CRCCP funding ends. Although there is a rapidly growing body of literature on sustainability [17–19], most of the studies are focused on the theoretical or methodological aspects of sustainability research. To date, only one study has examined the sustainability of the EBIs within the CRCCP [20]. Studies on sustainability as an outcome of intervention in various other settings have reported mixed results and evidence gaps [19, 21]. Given these evidence gaps and potential central role of sustained EBI implementation in achieving long-term impact of the CRCCP, this study aims to explore:

1. The proportion of clinics achieving EBI sustainability by specific EBI (e.g., client reminders, provider reminders), including how the four EBIs compare in terms of sustainability and whether trends are observed over time.
2. The factors associated with sustainability status (e.g., clinic and program characteristics).

We use an exploratory analysis and data driven approach to gain useful insights into the sustainability of CRCCP. This analysis approach is particularly appropriate for this inquiry, given the complex, multidimensional nature of sustainability and the current lack of standardized metrics and methodologies for its evaluation. By leveraging this explorative framework, this study aims to pave the way for hypothesis generation for future research and deepen our understanding of sustainability, ultimately contributing to the development of practical implementation strategies to enhance the long-term impact of public health interventions.

## Methods

### Study population and data

Our study population includes all partnering health system clinics active for at least one year in the CRCCP from 2015 through 2020. Clinics not implementing the specific EBI or with missing EBI implementation data were excluded before creating the final study sample. We used CRCCP Clinic Data which is collected by CDC for program evaluation. All CRCCP recipients report to CDC a baseline and annual clinic-level data record for each partner clinic. These data include information on clinic characteristics, program implementation, and clinic-level screening prevalence. The data reporting process is iterative; CRCCP recipients gather relevant data from clinics and enter or update using a web-based tool called CBAR. The data captures one record for each clinic but could be contributed by multiple individuals familiar with the clinic’s operations. This process also includes validation checks and addressing data quality issues using CDC provided technical assistance [7, 22]. Clinics may enter the program at any point during the 5-year project period and may exit before the program concludes. Therefore, the number of annual records submitted for clinics varies.

### Definitions of outcome variables

As part of the clinic record, CRCCP partner clinics report whether each EBI was in place (i.e., implemented) during the year and, if so, the sustainability of the EBI. The sustainability question includes the following response options: “sustainable without CRCCP resources,” “sustainable with CRCCP resources,” and “not sustainable”. According to CDC, an EBI is considered “sustained” when an EBI is fully integrated into clinic operations whereby high-quality implementation has been achieved and supporting infrastructure is in place, along with any financial support needed to maintain the EBI. Additionally, an EBI must be considered an institutionalized component of clinic operations. For this study, the EBI was determined “sustainable” if a clinic reported it as “sustainable without CRCCP resources”. Otherwise, the EBI was deemed not sustainable. Data on EBI sustainability is included, for each EBI, in the annual clinic data reporting.

We assessed EBI sustainability using two complementary approaches– cross-sectional and longitudinal–to present all relevant data. A *narrow* definition of sustainability was used to measure sustainability of EBIs for the clinic’s last year of program participation yielding a single data point per clinic for cross-sectional data analysis. For the narrow definition, clinics with only one year of program participation were excluded from the analysis, as it was assumed that the limited exposure to the program would not be sufficient to demonstrate sustainability. We also used a *broad* measure of sustainability to assess EBI sustainability for every year of clinic participation resulting in as many data points per clinic as the years of program participation for that clinic. The broad measure of sustainability resulted in a longitudinal data structure. We use the term “clinic year(s)” to represent each year of a clinic’s participation, regardless of the program year the clinic was enrolled. For instance, clinic year 1 represents the first year of program participation for a given clinic, regardless of when during the 5-year program period the clinic was enrolled in the program; clinic year 3 represents a clinic’s third year of program participation. If a clinic partnered with CRCCP for all 5 years of the program period, the clinic is counted as having 5 clinic years.

### Explanatory variables

Several variables from the Clinic Data were used as explanatory variables to assess EBI sustainability. Some of these variables characterized clinics as clinic type (e.g., Federally Qualified Health Centers, privately owned clinics), clinic size (e.g., small, large based on the number of screening eligible patients), and clinics’ location (e.g., metro, urban or rural). The classification of clinics’ location was based on U.S. Department of Agriculture’s rural–urban continuum codes [23]. Additional variables captured programmatic elements, including the implementation of the EBIs, other program components, and clinic’s participation in the CRCCP. These variables included whether clinics offered free fecal immunochemical testing kits (FIT kits), number of EBIs in place in the measurement year, whether the particular EBI was in place at baseline (i.e., before CRCCP), whether the EBI was newly implemented, whether the existing EBI was enhanced (i.e., by allocating CRCCP resources towards the EBI), and whether a screening champion, screening policy or patient navigation were in place during the last active clinic year. Because the data were collected at the clinic level, the clinic served as a unit of analysis. Patient data reported for the baseline and annual clinic records were aggregated (e.g., number of patients ages 50–75).

### Data analyses

We conducted descriptive analyses to examine frequencies, means and trends of the outcome variable, sustainability, as defined in the study. Logistic regressions were used to explore the association of multiple explanatory variables with EBI sustainability based on the narrow definition. The selection of explanatory variables was based on their availability in the Clinic Data, prior studies [7, 13, 24], and our prior assumption about their relevance to the outcome. All explanatory variables were used in the regression models initially; we then used the backward elimination method [25] to remove variables using a threshold of *p* = 0.10. The backward elimination method was planned beforehand to bring objectivity in variable selection, reduce noise and redundancies to identify key predictors, and reduce distortions in the regression estimates. The same logistic model was repeated for each EBI using the same set of explanatory variables. All analyses were conducted using SAS 9.4 software (SAS Inc., Cary, NC).

## Results

A total of 861 clinics met inclusion criteria, having participated in the program for at least one year. For the narrow definition of sustainability, we excluded 84 clinics because they had only one year of program participation. We also excluded those that did not implement a specific EBI in their final year, with 124 clinics for client reminders, 115 for provider reminders, 159 for provider assessment and feedback, and 223 for reducing structural barriers. As a result, the number of clinics analyzed using the *narrow* definition of the sustainability, ranged from 554 (reducing structural barriers) to 662 (providing provider reminders) (Table 1). For the *broad* definition of the outcome, all 861 clinics were included in the study sample with an average program participation of 3.62 years per clinic, resulting in a total of 3,116 clinic-years.

**Table 1:** Summary statistics of clinic characteristics and other analysis variables

	Client Reminders				Provider Reminders				Provider Assessment and Feedback				Reducing Structural Barriers		
	*N* = 653				*N* = 662				*N* = 618				*N* = 554		
Clinic type – frequency (%)															
- Federally qualified health centers	478		(73.20)		489		(73.87)		447		(72.33)		446		(80.51)
- Hospital/health system owned	92		(14.09)		90		(13.60)		92		(14.89)		46		(8.30)
- Private/physician owned	43		(6.58)		40		(6.04)		44		(7.12)		24		(4.33)
- Other (e.g., tribal health)	40		(6.13)		43		(6.50)		35		(5.66)		38		(6.86)
Clinic size – frequency (%)															
- Small (< 500 patients)	179		(28.19)		180		(27.91)		165		(27.32)		157		(29.02)
- Medium (500–1500 patients)	261		(41.10)		271		(42.02)		248		(41.06)		222		(41.04)
- Large (> 1500 patients)	195		(30.71)		194		(30.08)		191		(31.62)		162		(29.94)
Clinic location – frequency (%)															
- Metro (population more than 1 million)	445		(69.31)		448		(68.82)		411		(67.60)		393		(72.24)
- Urban (population 20,000–1 million)	156		(24.30)		161		(24.73)		158		(25.99)		119		(21.88)
- Rural (population up to 20,000)	41		(6.39)		42		(6.45)		39		(6.41)		32		(5.88)
EBI in place in the baseline – frequency (%)															
- Yes	392		(60.12)		479		(72.47)		356		(57.61)		307		(55.62)
- No	260		(39.88)		182		(27.53)		262		(42.39)		245		(44.38)
Count of other EBIs in place in the evaluation year – Mean (SD)	2.55		(0.70)		2.59		(0.61)		2.56		(0.77)		2.73		(0.55)
Champion in place in the last clinic year – frequency (%)															
- Yes	537		(82.24)		539		(81.42)		496		(80.26)		441		(79.60)
- No	116		(17.76)		123		(18.58)		122		(19.74)		113		(20.40)
Free FIT kit available – frequency (%)															
- Yes	248		(37.98)		246		(37.16)		235		(38.03)		231		(41.70)
- No	405		(62.02)		416		(62.84)		383		(61.97)		323		(58.30)
Count of Active years in the program – Mean (SD)	3.8		(1.16)		3.79		(1.15)		3.73		(1.17)		3.78		(1.19)
Year 1 EBI newly implemented – frequency (%)															
- Yes	192		(29.40)		186		(28.10)		138		(22.33)		163		(29.42)
- No	461		(70.60)		476		(71.90)		480		(77.67)		391		(70.58)
Year 1 EBI enhanced – frequency (%)															
- Yes	319		(48.85)		308		(46.53)		297		(48.06)		245		(44.22)
- No	334		(51.15)		354		(53.47)		321		(51.94)		309		(55.78)
Patient navigator in place in the last CY – frequency (%)															
- Yes	310		(47.47)		305		(46.07)		263		(42.56)		282		(50.90)
- No	343		(52.53)		357		(53.93)		355		(57.44)		272		(49.10)
Policy in place in the last CY – frequency (%)															
- Yes	605		(92.65)		611		(92.30)		564		(91.26)		514		(92.78)
- No	48		(7.35)		51		(7.70)		54		(8.74)		40		(7.22)

*Abbreviations*: *EBIs* Evidence Based Interventions, *FIT* Fecal Immunochemical Test, *SD* Standard Deviation, *CY* Clinic Year

Table 1 shows the summary statistics of the study variables. Sample size across the EBIs varied because the prevalence of implementation varied by EBI. Most clinics were FQHCs (> 70%) and were located in Metro areas (about 70%), but the clinics were more evenly distributed by size. The baseline implementation EBIs varied, with 55.62%–72.47% of clinics having an EBI in place. On average, clinics had 2.55–2.73 EBIs active during the evaluation year in addition to the EBI in question. The presence of clinic champions was high, with about 80% of clinics reporting champions in the measurement year, while free fecal immunochemical test (FIT) kit availability ranged from 37.16% to 41.70%. Clinics had an average of 3.73–3.80 active years in the program. More than one fifth or 22.33%–29.42% of clinics newly implemented an EBI in the first year, while 44.22%–48.85% enhanced an EBI. Patient navigation services were reported in 42.56%–50.90% of clinics, and over 91% of clinics had a policy in place during the evaluation year, i.e., their last clinic year.

Table 2 shows the sustainability of EBIs based on the narrow definition. About 82% of the clinics reported provider reminders as sustainable, followed by provider assessment and feedback (70.71%), and client reminders (58.96%). Just over half (55.60%) of clinics reported reducing structural barriers (i.e., non-economic obstacles such as limited clinic hours) as sustainable.

**Table 2:** Number of clinics reporting sustainability of EBIs without CRCCP resources in the last clinic year

EBIs	Sustainability (narrow definition)	
	Number of clinics (n)	Yes (%)
Client reminders	653	58.96
Provider reminders	662	81.72
Provider assessment and feedback	618	70.71
Reducing structural barriers	554	55.60

*Abbreviations*: *EBIs* Evidence based interventions, *CRCCP* Colorectal Cancer Control Program

**Fig. 1:**
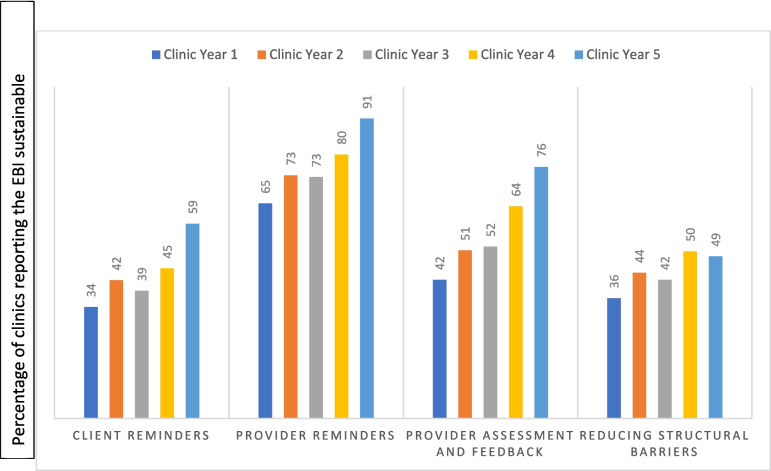
Sustainability trend: by EBI. *Abbreviations*: EBIs: Evidence based interventions, CRCCP: Colorectal Cancer Control Program

Based on the broad definition, we examined the trends of sustainability for each EBI from a clinic’s first year in the program to their last year in the program, with a maximum of 5 years as seen in Fig. 1. The percentage of clinics able to sustain each of the four EBIs trended upwards over time. Improvement in EBI sustainability over time was greatest for provider assessment and feedback with a 34-percentage point increase. The sustainability of reducing structural barriers had the smallest gain over time among the four EBIs with a 13-percentage point increase. Provider reminders had the highest sustainability over the years, increasing from 65% in Year 1 to 91% in Year 5.

Next, we examined the distribution of sustainability categories using the broad definition (Table 3). Starting from the bottom, the"EBI not in place” category represents clinics that did not yet have the EBI implemented, making the sustainability question inapplicable. The “not sustainable” category reflects that the EBI was in place, but not sustainable, with or without the use of CRCCP resources. Even with CRCCP support, some clinics did not report the EBIs as sustainable. The proportion of clinics that reported sustainability of an EBI without CRCCP resources ranged from 28.98% clinic years for reducing structural barriers, 32.32% for client reminders, 39.92% for provider assessment and feedback, and 59.4% for provider reminders. The remaining sustainability category represented clinics reporting EBIs as sustainable, but only with CRCCP resources (e.g., technical support, funding). Depending on the EBI, the range was from about 16% to 40% of clinic years.

**Table 3:** Measure of sustainability over all the clinic years (broad definition) (*N* = 3,116)

Sustainability categories	Sustainability (all active clinic years)							
	Client reminders *n* = 3116		Provider reminders *n* = 3116		Provider assessment and feedback *n* = 3116		Reducing structural barriers *n* = 3116	
	Freq	%	Freq	%	Freq	%	Freq	%
Sustainable, without CRCCP resources	1007	32.32	1851	59.4	1244	39.92	903	28.98
Sustainable, with CRCCP resources	1249	40.08	490	15.73	893	28.66	1076	34.53
Not sustainable	167	5.36	169	5.42	205	6.58	111	3.56
EBI not in place	693	22.24	606	19.45	774	24.84	1026	32.93

*Abbreviations*: *EBIs* Evidence based interventions, *CRCCP* Colorectal Cancer Control Program

Lastly, we ran regression models for the narrow definition of the outcome variable, the sustainability of each EBI in a clinics’ last year of program participation (Table 4). The initial model used all variables from the Table 1. The backward elimination method dropped one or more variables from the final model, and dropped variables are indicated by blanks (–) in the table cells. Results show that the odds ratios for sustainability are lower in clinic types other than FQHCs, which are the reference category in the model. Medium sized clinics (vs. small clinics) and metro clinics (vs. urban clinics) had lower odds ratios for sustainability.

**Table 4:** Regression results of the relationships between sustainability and various clinic and programmatic factors by EBIs

**Explanatory variables**	Client reminders			Provider reminders			Provider assessment and feedback			Reducing structural barriers		
	**Odds Ratio**	**95% CI**		**Odds Ratio**	**95% CI**		**Odds Ratio**	**95% CI**		**Odds Ratio**	**95% CI**	
**Clinic type**												
- Federally qualified health centers	Ref			Ref			Ref			Ref		
- Private	**0.048**	0.017	0.135	**0.168**	0.076	0.371	**0.085**	0.039	0.187	**0.228**	0.084	0.618
- Hospital	**0.455**	0.253	0.818	–	–	–	–	–	–	0.504	0.239	1.065
- Other	**0.116**	0.049	0.274	–	–	–	**0.141**	0.059	0.339	**0.373**	0.151	0.921
**Clinic size**												
- Small	Ref			Ref			Ref			Ref		
- Medium	**0.629**	0.418	0.946	–	–	–	0.662	0.433	1.010	**0.616**	0.404	0.939
- Large	–	–	–	–	–	–	–	–	–	–	–	–
**Clinic location**												
- Urban	Ref			Ref			Ref			Ref		
- Metro	**0.494**	0.304	0.801	**0.402**	0.221	0.732	**0.388**	0.232	0.652	**0.460**	0.282	0.752
- Rural	–	–	–	–	–	–	–	–	–	–	–	–
Baseline – EBI in place (= yes)	**12.300**	4.616	32.776	**24.409**	9.679	61.553	**2.408**	1.139	5.094	**9.570**	4.608	19.873
Number of other EBIs in place in the evaluation year (0—3)	**3.562**	2.606	4.869	**1.627**	1.095	2.419	**3.486**	2.535	4.793	**2.003**	1.362	2.947
Champion in place in the last clinic year	**2.883**	1.652	5.031	**2.525**	1.481	4.305	–	–	–	**2.284**	1.337	3.902
Free FIT kit available (= yes)	–	–	–	**3.087**	1.827	5.215	**2.149**	1.337	3.453	**1.940**	1.244	3.025
Active years in the program (Years)	–	–	–	**1.610**	1.304	1.988	**1.316**	1.097	1.578	–	–	–
Year 1 – EBI newly implemented (= yes)	–	–	–	**28.083**	10.071	78.312	–	–	–	–	–	–
Year 1 – EBI enhanced	**0.149**	0.057	0.391	–	–	–	**0.368**	0.178	0.761	**0.154**	0.076	0.310
Patient navigator in place in the last CY	**0.183**	0.117	0.286	–	–	–	**0.419**	0.263	0.667	**0.130**	0.079	0.212
Policy in place in the last CY	–	–	–	**0.248**	0.075	0.825	–	–	–	–	–	–

*Abbreviations*: *EBIs* evidence-based interventions, *CRCCP* Colorectal Cancer Control Program, *CI* confidence interval, *FIT* fecal immunochemical test, *CY* Clinic Year, *PN* patient navigator

There were two most significant predictors of sustainability for all four EBIs. The first was whether the EBI was in place at the time of clinic’s recruitment in the program (i.e., baseline). Each EBI, if it was in place at baseline, had a higher odds ratio for sustainability. The second important predictor was the number of other EBIs in place in that year (i.e., last year of participation). The odds ratios for sustainability of any EBI were higher if there was one additional EBI in place in that year. Note that clinics could have up to four EBIs in place at any given year. The third important predictor was screening champions. Clinic sustainability of all EBIs other than provider reminders were positively associated with the presence of a screening champion. Similarly, the availability of free fecal screening kits was a significant predictor of the sustainability of all EBIs except client reminders. Length of clinic participation (i.e., number of active years in the program) was associated with sustainability for two of the four EBIs, i.e., provider reminders and provider assessment and feedback. If provider reminder was newly implemented in clinic year 1, the EBI had an odds ratio of 28.1 for sustainability. However, this was not true for other EBIs. For three of the predictor variables, which include enhancement of the EBI in Program Year 1, patient navigation and screening policy, the odds ratios were not favorable for sustainability for one or more of the EBIs.

## Discussion

Sustaining effective public health interventions, which is an integral part of implementation strategies, is critical to ensure lasting impact and maximize the return on investment. CRCCP is one of CDC’s flagship cancer control programs and has been shown to increase screening prevalence in clinics that serve people from under-resourced communities, also reducing cancer-related disparities. CDC established sustainability as a tenet of the CRCCP with the goal of integrating and institutionalizing EBIs within clinics [7]. The CRCCP’s health system change approach, with a focus on implementing and sustaining EBIs, was a departure from an earlier model where screening services were provided directly to eligible patients using program dollars [7]. In this sense, the novel CRCCP approach has the potential to reach greater numbers of patients with a sustainable program model. Furthermore, the continued sustainability of EBIs is a critical factor in maintaining or further augmenting screening prevalence in the long term.

As far as we know, this study is among the first to examine EBI sustainability of cancer screening interventions in health clinic settings. We analyzed CRCCP Clinic Data to explore some basic questions based on clinics’ self-assessment of EBI sustainability. We employed an exploratory approach in our analysis and presented all available sustainability related information in the data. Our study found that sustainability varied widely by EBI. After five years of program participation, over 90% of clinics reported provider reminders as sustainable, which is likely facilitated through their integration into clinic electronic health record (EHRs) systems. Of interest, while most clinics reported provider reminders as sustainable, earlier CRCCP studies have found it to be less effective in increasing CRC screening prevalence [13, 14, 24]. Randomized controlled trials studying the impact of provider reminders alone, not as part of multi-component intervention, have also reported that it is not significantly associated with increased screening rate [26–28]. Therefore, it is possible that providers may not be heeding and/or patients may not be responding to the provider reminders. This example highlights the fact that EBI sustainability alone may not be the only contributor to increases in screening prevalence. The reach, effectiveness, and sustainability all influence the longer-term impact of the intervention. We plan to study why provider reminders have had a limited effect in the CRCCP.

Provider assessment and feedback were found to be another sustainable EBI. Many EHR systems are programmed to generate provider reports, suggesting a one-time or periodic investment of resources may be adequate to integrate and automate this EBI. Similar to provider reminders, sustainability of this EBI might have been linked to incentives or accolades for providers, which we plan to investigate further.

In contrast, reducing structural barriers was reported as the least sustainable EBI. This strategy represents a wide range of approaches such as providing patient navigation, transportation and translation services, extending clinic hours, providing pre-paid mail back materials for fecal blood tests, and providing childcare. Most of these require on-going resource allocation that may hinder sustainability. Similarly, client reminders are more difficult to automate and may also demand more resources, depending on the approach taken by clinics (e.g., personal phone calls, letters or post cards, text messages).

Overall, a large proportion of clinics, in terms of clinic years, reported that the EBIs were sustainable with continued CRCCP resources, and a small portion of these (from 3.6%—6.6%) reported that they could not sustain EBIs regardless of the availability of resources. This suggests that, for a significant number of clinics, sustainability depended upon the continued availability of CDC funds. This implies that, in the long run, the lack of sustainability may be attributed to a funding gap, which was temporarily filled by the CRCCP. This gap was most significant for client reminders and the least for provider reminders. Continued availability of funding is also identified as one of the most critical factors for the sustainability of a public health program [19, 29].

Notably, sustainability increased over the five-year program period for each EBI as shown by their upward trends with the greatest increase observed for provider assessment and feedback. The fact that EBIs became more sustainable over the five-year period may be attributed to the clinics’ participation in the CRCCP, including the investment of resources made by the program. Factors associated with sustainability show that EBI’s sustainability was greater if the EBI was in place at baseline and the clinic had other EBIs in place. Implementing multiple EBIs may reflect a clinic’s commitment to implementing EBIs and achieving their sustainability. Of note, screening champion was also associated with greater rate of sustainability for three of the four EBIs. Previous studies of the CRCCP have found champions to be an important and most consistent driver of the increase in screening prevalence in clinics [7, 13, 14]. Therefore, champions appear to play a critical role in achieving two crucial program outcomes in the CRCCP. CDC has a study underway looking more closely at program champions and their role in the CRCCP. Finally, free fecal blood test kits were associated with greater sustainability for three of four EBIs. These results imply that sustainability is largely linked with resources—financial or non-financial resources such as staff capacity, leadership support, champions, etc. We found that for all four EBIs, sustainability increased with the availability of CRCCP resources. This suggests that the CRCCP is temporarily filling a critical resource gap, enabling clinics to keep or add EBIs that may be sustained in the long run. On the other hand, clinics using patient navigators were less likely to report sustainability of any EBI. This may be attributed to the scenario in which clinics use patient navigators to implement EBIs (e.g., the navigators administering client reminders) and patient navigators themselves are not sustainable due to the lack of funding availability in the long term. The sustainability gain over the period of 5 years can be attributed to the CRCCP intervention, and it is likely to continue at least partially even after the program support ends. Assessing longer term EBI sustainability after the CRCCP ceases to support clinics, is an area for future study. Relatedly, assessing whether EBI sustainability does, in fact, help maintain screening prevalence increases is another area of future research.

Based on our results, some EBIs may need a one-time or periodic investment, while others require the continuous investment of resources. A qualitative study of the CRCCP found that EBI sustainability was improved by certain measures, such as providing some financial resources, integrating EBIs into existing clinic workflows and EHRs, and engaging care teams and clinic leadership [30]. Where possible, automating EBI implementation through their integration in EHRs and workflows may be particularly effective and efficient to achieve sustainability [31, 32]. Promoting sustainability during the planning process is another strategy that could be valuable. CDC requires CRCCP award recipients to conduct readiness assessment of potential clinic partners. This process can include explicit planning for EBI selection and implementation to maximize sustainability and may benefit from having leadership support early in the process [33, 34].

Our study is exploratory and data-driven, rather than guided by a specific theory or hypothesis. Nonetheless, the key insights generated align closely with established frameworks that conceptualize implementation strategies. For instance, the sustainability framework developed by Schell et al. [29] identifies nine core domains that influence a program’s capacity for sustainability. Several of these domains—such as funding stability, organizational capacity, political support, program adaptation, and evaluation—emerged in our study as critical enabling factors for sustained implementation [29]. Furthermore, in today’s rapidly evolving healthcare landscape and across diverse clinical and community settings, the sustainability of public health interventions should be understood as an ongoing, adaptive process. A dynamic sustainability framework suggested in the literature recognizes the importance of continuously assessing and responding to changes in the context of care delivery to refine and enhance interventions over time [35]. Tools and guidance for sustainable EBI implementation are becoming increasingly available. The Field Guide for Assessing Readiness to Implement Evidence-based Cancer Screening Interventions provides guidance in planning and preparedness for sustainable implementation of the program [33, 36]. Recipients in the CRCCP have developed notable tools such as Program Sustainability Assessment Tool to help implementation partners [37].

### Limitations

The study has several limitations. The definition of EBI sustainability used in the study is limited in scope and based on self-reporting by the partnering clinics. The definition did not directly take into consideration the sustainability of increased screening prevalence, the primary outcome of the CRCCP intervention. The available data only supported analysis for the 5-year program period – data beyond the 5-year window could measure sustainability more robustly and accurately. The data used only captured whether an EBI was implemented and sustainable and it did not have any information on the quality of EBI implementation which could impact the effectiveness of the EBIs implemented. Our exploratory analysis suggests that many factors explain the sustainability of EBIs, however other potential factors (e.g., clinics’ resourcefulness, priorities) that also determine sustainability were not available in our data, and hence missing from the analysis. We are unable to determine if there is any bias in the regression results due to missing variables.

## Conclusions

Sustainability of interventions introduced by public health programs like the CRCCP is critically important in optimizing the allocation of limited public health and clinical resources. The Community Guide recommended implementation strategies studied here are evidence based, but their actual and long-term success will also depend on their sustainability. We found substantial improvement in the sustainability of EBIs over the 5-year program period, although results varied by EBIs and room for improvement remains. The outcomes of this investigation offer valuable insights for future implementation of EBIs, guiding the prioritization of EBIs characterized by greater sustainability and facilitating an exploration of the factors (e.g., champions) contributing to the differential sustainability of various interventions. Future studies can investigate EBI sustainability after a clinic’s participation in the CRCCP to better assess the long-term impact of this program and examine whether EBI sustainability is associated with maintenance, or even improvement, of screening prevalence.

## Data Availability

The datasets generated and/or analyzed during the current study are not publicly available due to its specific nature and purpose of use. These data in limited form may be available from the CDC for research purposes if such a request is made.

## Abbreviations

CDC: Centers for Disease Control and Prevention
CRCCP: Colorectal Cancer Control Program
CRC: Colorectal Cancer
EBI: Evidence Based Intervention
USPSTF: The United States Preventive Services Task Force
FQHC: Federally Qualified Health Centers
FIT: Fecal Immunochemical Test
HER: Electronic Health Record
